# Commentary: endoscopic vacuum therapy for anastomotic leakage after esophagectomy and total gastrectomy: obstacles to finding true evidence

**DOI:** 10.1093/dote/doab023

**Published:** 2021-04-24

**Authors:** Sander Ubels, Moniek Verstegen, Stefan Bouwense, Bastiaan Klarenbeek, Frans van Workum, Camiel Rosman

**Affiliations:** 1 Department of Surgery, Radboud University Medical Center, Nijmegen, The Netherlands; 2 Department of Surgery, Maastricht University Medical Center+, Maastricht, The Netherlands; 3 Department of Surgery, Canisius-Wilhelmina Hospital, Nijmegen, The Netherlands

Dear Editor,

We read with great interest the systematic review and meta-analysis by Tavares et al. which aimed to provide ‘best evidence’ by evaluating the safety and efficacy of vacuum therapy and comparing vacuum therapy to stent treatment for anastomotic leakage.^1^ The main findings were a higher closure rate and lower rate of mortality in favor of vacuum therapy compared to stent treatment. The authors assign a ‘moderate’ certainty of evidence to this finding.

In our view, interpretation of comparative analyses on anastomotic leakage in currently available literature is problematic for three reasons. First, most studies report very little leakage characteristics (e.g. mediastinal/pleural contamination or leakage circumference) and patient parameters at leak diagnosis (e.g. clinical condition or presence of organ failure). However, these parameters may be crucial to understand the wide-ranging clinical presentation of anastomotic leaks. In addition, these parameters affect treatment decisions, and thus need to be corrected for during comparative analyses to reduce confounding bias. Second, the fact that the treatment strategy of anastomotic leakage is usually a combination of multiple interventions is often overlooked by studies focusing on a specific treatment modality. Whilst endoluminal vacuum therapy by itself may be viewed as a strategy (i.e. combining drainage and defect closure), stent treatment is rarely a complete treatment strategy. Additional interventions (e.g. drainage of fluid collections) performed synchronously for management of anastomotic leaks are often not reported. Third, selection of patients in current studies may complicate interpretation of comparative analyses. For example, the largest study included by the meta-analysis introduced selection bias by excluding patients who underwent surgical treatment after failure of endoluminal vacuum therapy or stent treatment.^2^ Taken together, it is unknown whether patients and cohorts are truly comparable, due to insufficient detail in reporting, and results of comparative analyses are probably affected by selection and confounding bias.

The authors used the proper scientific tools to assess the risk of bias and quality of evidence of the included studies.^3^^,^^4^ However, the grading of selection-, confounding-, and overall bias as low for the majority of studies may have been too optimistic. Therefore, we believe that the findings and implications of the study by Tavares and colleagues should be interpreted in perspective of the limitations of the current body of literature on anastomotic leaks. Authors of other studies have formulated their conclusions more cautiously after recognizing the limitations of the current studies on treatment of anastomotic leaks.^5–7^

The search for more evidence on anastomotic leakage treatment efficacy continues and the scientific community is eagerly awaiting evidence supporting a specific treatment for anastomotic leakage in order to improve outcomes of leak treatment. A tool to correct for leakage characteristics and patient parameters at time of diagnosis of the leak is currently being developed and may be used to reduce the risk of confounding bias in future studies.^8^ We believe that improving detailed reporting of leakage characteristics and treatment procedures will increase the quality of studies and promotes finding true evidence that is essential to improve outcomes of patient with anastomotic leakage after esophagectomy and total gastrectomy.
